# Economic and environmental effects of unilateral climate actions

**DOI:** 10.1007/s11027-014-9597-9

**Published:** 2014-07-09

**Authors:** Olga Kiuila, Krzysztof Wójtowicz, Tomasz Żylicz, Leszek Kasek

**Affiliations:** 1 Faculty of Economic Sciences, University of Warsaw, 44/50 Dluga, Warsaw, 00-241 Poland; 2 Ministry of Economy, Strategy and Analyses Department, Warsaw, Poland; 3 World Bank, Warsaw, Poland

**Keywords:** Carbon leakage, European union policy, Computable general equilibrium modeling, Decomposition analysis

## Abstract

Unilateral climate policy can be detrimental to global climate protection. Our objective is to provide insight into such a policy, to quantify the risk of carbon leakage, and to investigate the effects related to potential anti-leakage measures. We analyze existing definitions of carbon leakage and propose an alternative, rigorous one, which is different in three respects. The definition is then tested using computable general equilibrium analysis of the global economy and decomposition analysis. We identify a list of parameters that affect not only the magnitude but also the sign of the carbon leakage rate. Manipulating elasticities of substitution suggests that carbon leakage can be either positive or negative. Computable general equilibrium models, which are widely applied, including by the European Commission in this area, should be transparent, and their assumptions call for careful validation. We find that emission limits are properly distributed between sectors covered by the European Union Emissions Trading System and other sectors for the first commitment period (ended in 2012) but not for the second one (ending in 2020), where the target for the non-trading sectors should be reduced relative to the target for the trading sectors in order to equlize marginal abatement costs.

## Introduction

The aim of the paper is to analyze the economic and environmental consequences of a unilateral policy for the abatement of a global pollutant. To this end, we define the concept of leakage, apply appropriate simulation models, decompose the expected emission changes, and carry out sensitivity analyses. One controversial issue regarding how to protect the climate is whether non-global abatement commitments can reduce global carbon dioxide (CO_2_) emissions. With the help of a computable general equilibrium (CGE) model, we demonstrate that the answer is far from obvious and that it depends on technical assumptions that have been insufficiently studied to date.

The economic impacts of mitigation strategies and regional burden-sharing have been the focus of many analyses of climate action, particularly analyses of unilateral reduction strategies adopted or to be adopted by the European Union (EU) (see Steininger et al. 2011, Boehringer et al. 2010, Mechler et al. 2010, Kuik 2003). This paper provides a similar exercise but with important value added, as briefly explained below. Namely, we propose a new, rigorous definition of carbon leakage, we provide a decomposition of the change in carbon emissions into four effects, and we simulate a clean development mechanism in two alternative ways. Our goal is to provide an economic analysis of unilateral climate policy using the example of the EU, quantify the risk of carbon leakage, and investigate the effects related to potential anti-leakage measures. Our hypothesis is that unilateral climate policy is ineffective and may even be detrimental to global climate protection.

The Berlin Mandate (1995), under the United Nations Framework Convention on Climate Change (UNFCCC), establishes 'common but differentiated responsibilities’. One group of countries (Annex I countries) is to take binding commitments, whereas the rest of the world (non-Annex I countries) is not required to take any. A major effect of the Berlin Mandate was the decision by the USA (an Annex I country) not to join the UN FCCC Kyoto Protocol (1997). Other countries from Annex I (such as Canada, Russia, and Japan) indicated that they would not take up targets in the second commitment period (2013–2020), even though they enlisted for the first commitment period (2008–2012). The EU, as the only top-emitting member, has enlisted for the second commitment period and has even tried to increase the target for itself unilaterally.

We use a global CGE model to perform a simulation analysis for the year 2020. A major issue in modeling exercises of carbon leakage is that they reflect the authors’ assumptions regarding actions that are expected on behalf of some agents, whereas these actions may crucially depend on agreements reached and instruments applied. We demonstrate that assumptions widely accepted in economic analyses drive the results of models serving as a basis for policy inspiration.^1^

Another area where modeling can strongly influence policies is scenario building. Frequently, scenarios rely on hypothetical actions that reflect analysts' expectations or convictions rather than realistic projections. An example of an approach that stresses the need for achieving certain outcomes rather than studying which decisions are likely to solve the problem is provided by Van Vuuren et al. (2011) and Heindl and Voigt (2012). In this vein, we analyze certain questions regarding climate protection through a clean development mechanism (CDM) scenario. European legislation allows domestic firms to comply with some requirements using offsets that are validated by external parties. In this regard, doubts arise because reductions refer to baseline paths that are not binding for the host countries. Our simulation considers two cases, where the baseline emission level for non-Annex I countries is determined before and after the mechanism is implemented. The rationale behind introducing CDM is lowering the abatement cost, which might help reduce leakage. The first case (which does not apply when non-Annex I countries lack binding emission ceilings) solves the problem associated with carbon leakage, but in the second one, we observe a significant increase in carbon leakage.

## Carbon leakage and anti-leakage measures

Carbon leakage (CL) is commonly defined as an emission in one geographical area resulting from a decrease in emissions elsewhere, everything else being constant, including policies applied elsewhere. It is possible that these policies may be altered as a result of inspiration by a certain region's unilateral reduction policy. Let N denote the region toward which carbon emissions leak to, even though the region may undertake certain climate-protection actions, and let A denote the region that undertakes a more ambitious abatement program. One approach is to define CL as the difference between the expected emissions in region N if there is an abatement program in region A and the emissions in region N, provided business-as-usual (BAU) policies in region A:

1 $$ \mathrm{CL}\left(\varDelta \mathrm{R}\right) = \left({\mathrm{f}}_{\mathrm{N}}\left({\mathrm{GDP}}_{\mathrm{N}},{\mathrm{P}}_{\mathrm{N}},{\mathrm{GDP}}_{\mathrm{A}}\left({\mathrm{R}}_0+\varDelta \mathrm{R}\right)\right)-{\mathrm{f}}_{\mathrm{N}}\left({\mathrm{GDP}}_{\mathrm{N}},{\mathrm{P}}_{\mathrm{N}},{\mathrm{GDP}}_{\mathrm{A}}\left({\mathrm{R}}_0\right)\right)\right)\ /\ \varDelta \mathrm{R} $$

where R_0_ is the baseline reduction target adopted in A, ∆R is an additional reduction target contemplated in A, P_N_ indicates an abatement policy adopted in N, f_N_ is an emission function for N, and GDP_A_ (Gross Domestic Product in A) is a function of a reduction target adopted in A. GDP may not be included in the CL formula directly, but it represents an economic situation determined by a given scenario. This definition of carbon leakage allows for any sign and any level of the indicator. Thus, CL(∆R) can be either positive or negative.^2^ In our definition, it is crucial that only R and GDP_A_ change. However, it is very difficult to comply with this assumption because all variables are linked to each other. The GDP and emissions in each region depend on the reduction targets in several ways. First, increasing reduction targets is typically associated with a slowdown in GDP growth. Second, increasing reduction targets in A is expected to decrease global prices of fossil fuels, which is likely to increase the demand for fossil fuels and the emissions in N. However, this effect is not observed in regions with binding commitments. Third, technological progress and economies of scale in low-carbon technologies are likely to drive costs down (both in A and N). Fourth, changes in GDP imply changes in trading patterns between A and N. Consequently, it is difficult to ensure that CL(∆R) indeed complies with theory.

If carbon leakage were to be attributed to the complexity of the global economic system and myriad other factors, then relating CL to changes in climate policies would be difficult. A carbon leakage defined in a broader sense deserves further research, but it is beyond the scope of our paper. We consider a narrower definition of CL, in which the only source of leakage is a shift from the current carbon reduction target to a more ambitious target. Our definition differs from that provided in the relevant literature in three ways. First, a region N is usually defined as a region that undertakes no climate action. We stress that not all countries that undertake some climate action are qualified as a region A. A proper distinction between countries qualified as regions A and N is essential to determining the level of CL. Second, when several assumptions about scenarios are changed simultaneously, it is difficult to determine which one causes a change in CL. We identify a single change within each scenario to compare the CL rates among alternative simulations. Third, most authors use a BAU scenario with no climate action as a baseline to define CL. We assert that in baseline scenarios, a climate action should be included in the case in which region A has already taken some action.

CGE models reported in the literature imply very different CL rates. One way to explain these differences is to examine the elasticity assumptions. Gerlagh and Kuik (2007) carried out a meta-analysis of the literature and estimated an econometric model that establishes a link between elasticity parameters and CL. However, the model cannot be considered particularly satisfactory because the authors assumed a production function, which by definition assumes the leakage rate is less than 100 %. Thus, for instance, the authors did not include a study by Babiker (2005) in which a higher CL rate is obtained because increasing returns to scale are assumed for energy-intensive sectors. Even without the increasing returns, the product homogeneity assumption drives the CL rate up. Therefore, it is not surprising that the combination of constant returns to scale and product differentiation implies a low leakage rate.

Some authors indicate that the CL rate depends on which countries belong to the abating (A) region, but conclusions may differ. In particular, whereas an OECD (Organization for Economic Cooperation and Development) simulation (Burniaux et al. 2009 shows that CL should not exceed 6 % when all of Annex I is included in the coalition, Winchester (2011) obtains a 25 % carbon leakage rate. Boehringer et al. (2010) determined the leakage rate to be 10 % in the case of a unilateral US policy, 25 % in the case of a unilateral EU policy, and 15 % if the EU and the US undertake joint action. The lowest CL rate of 1 % was obtained by Mattoo et al. (2009). Unilateral commitments by a single region have also been analyzed in several other papers.

Some authors emphasize that the leakage rate depends on the level of abatement ambition. As a rule, the higher the level of abatement is, the higher the CL becomes, but this is not a universal finding. Bossello et al. (2011) obtained leakage rates of 74 % and 70 % when 20 % and 30 % GHG reduction targets were considered, respectively, for a unilateral EU mitigation policy (i.e., a higher carbon leakage corresponds to a lower emission target). In addition, Steininger et al. (2011) found that as climate policies become more stringent and comprehensive, the level of carbon dioxide before CO_2_ emissions in non‐abating regions decreases. A typical pattern found in the CGE literature is consistent with the results of Carbone et al. (2009), which indicate that the leakage rate is approximately 50 % when goods are homogenous across regions and over 20 % otherwise. Trade spillovers decrease global emissions whenever traded goods are imperfect substitutes.

Important aspects of leakage modeling include assumptions about the cost of climate mitigation. The lower the cost is, the lower the CL rate becomes (other things held constant). Beckman et al. (2009) explain why estimates of costs used in CGE models are likely to be too low, which is mainly due to overstating the price elasticity and elasticity of substitution. If elasticities are high, then substituting carbon-intensive factors is cheaper than it is in reality. Using values that are in line with the literature estimates, the authors found that the marginal abatement costs for greenhouse gases (GHGs) were underestimated by 57 %. This finding suggests that carbon leakage is grossly underestimated.

Numerous studies suggest that the carbon leakage rate is below 100 %, which means that unilateral abatement does contribute to climate protection. The global cost of abating a unit of carbon is higher than the cost that would be incurred under a hypothetical global agreement. This higher cost is incurred because the avoided emissions in A would need to be divided by 1-CL(∆R). On the other hand, carbon leakage may reach rates above 100 %, in which case unilateral abatement would be detrimental to climate protection.^3^

Historical records indicate that global GHG emissions have been growing over recent decades EBRD (2011) despite unilateral abatement actions undertaken by some regions since the 1990s. Non-Annex I countries do not report their carbon dioxide emissions under the UNFCCC. Thus, it is difficult to assess true global emissions. A challenge in the design of a unilateral climate policy is developing an appropriate response to the threat of carbon leakage. Anti-leakage policies have little effect on global welfare, but they might have significant effects on the EITE (energy-intensive, trade-exposed) sectors. The following anti-leakage measures have been contemplated.

To date, the implementation of a cap-and-trade scheme in the EU has been accompanied by allocating free allowances based on historical emissions (i.e., a grandfathering scheme), irrespective of current or future output. An alternative measure is an output-based allocation (OBA scheme) of emission permits, in which the allocation of free allowances is linked to and updated based on recent output. With this instrument, emitters are not forced to reduce emissions by decreasing production, but they have a motivation to reduce their carbon intensity. Second, there are measures for equalizing the cost of carbon for foreign competitors, embodied in the production cycle of tradable goods. Such measures are carried out by border tax adjustments (BTA), which usually require that importers purchase emission permits based on the carbon content of the imported goods. Alternatively, a border adjustment measure could provide rebates to exported goods to ensure competitiveness on global markets.

Even in a world where countries only pursue their national self-interest, an international system of tradable emission permits can achieve substantial emission reductions (Carbone et al. 2009). The currently applied CDM is regarded as an outreach to non-Annex I countries. However, the fact that host countries do not take any binding commitments raises questions regarding to what extent such policies contribute to global CO_2_ emission abatement. Effective carbon offsets could lower the carbon price in Annex I countries because GHG reduction efforts would be undertaken in places where they are most cost-effective and thus mitigate the risk of CL. However, given no binding commitments in non-Annex I countries and unclear baseline emissions paths, an ill-structured offsetting mechanism may lead to higher carbon dioxide emissions in developing countries.

Previous studies have accepted that conditions may change in regions with a non-abating policy, whereas we assume that there are no policy changes in those regions. We would like to answer the question concerning whether a unilateral emission reduction in region A (the EU in our model) can significantly reduce global emissions.

## Model and scenarios

Our simulation experiment starts with the global CGE model.^4^ The model is a static multi-sector, multi-region model based on the GTAP7 (Global Trade Analysis Project, version 7) database (Narayanan and Walmsley 2008), with the 2004 benchmark period recalibrated for 2020. The model incorporates market distortions (such as the existence of initial taxes) and market imperfections (such as labor market rigidities) that may change the costs of carbon abatement. Production technologies in all sectors are described in a conventional manner with a nested CES (constant elasticity of substitution) function using capital, labor, and energy as production factors. Global coverage of international trade and energy use across three regions, the EU, other industrialized economies (A1), and developing countries (DC), enables the analysis of international spillovers and feedback from climate policies on global energy prices.

The original 57 GTAP sectors were grouped into 13 sectors: 5 EITE sectors (chemicals, non-metallic minerals, iron-steel, non-ferrous metals, and paper-pulp-print), 5 energy sectors (coal, gas, crude oil, refined oil, and electricity), 2 transportation sectors (aviation and other transport), and other manufacturers and services including renewables. Only two of those sectors are not covered by the EU ETS (European Trade System): other transport and other manufacturers. In this context, coal is defined as hard coal, lignite and peat but does not include coke. Coke is included in refined oil (OIL) together with petroleum products and nuclear fuels (OIL is not included in the EITE sectors in the model to treat all fuels similarly). A crude oil (CRU) is not a direct source of carbon emissions. Furthermore, gas is defined as natural gas, whereas electricity and heating are aggregated together. The final demand consists of representative households, the government, and the investment sector. Households’ expenditures are described by a nested CES function. Governments earn income from taxes on producers, goods, and production factors. Those governments that adopt climate policies also gather revenues from selling CO_2_ rights. Investments are exogenous and are subtracted from households’ income.

The model’s horizon stretches to 2020, which is the deadline for the EU '20-20-20' package obligations. Trade is specified following the Armington approach (i.e., product heterogeneity of domestic and foreign goods). The values of elasticities are taken from the GTAP-E model (energy-environmental version of the GTAP model, McDougall and Golub 2007). Carbon emissions are linked in fixed proportions to the use of fossil fuels, which have different carbon contents. Carbon reduction occurs either by a fuel switch (limited substitution) or energy savings (reduction in economic activity). Non-CO_2_ emissions of GHGs are not considered. The only channel of carbon emissions in the model is the combustion of fossil fuels. We investigate how the carbon leakage rate changes when

- the EU adopts more ambitious targets without a comparable effort in other regions (REF and HIGH scenarios)
- compensating measures are introduced to protect domestic producers (OBA and BTA scenarios), and
- developing countries participate in the climate action through international offsets (CDM and CDM_NEW scenarios).

Policy scenarios are compared with the BAU scenario that assumes the economic and environmental forecast for 2020, in line with the base case considered by the International Energy Outlook (EIA 2009).^5^ The reference (REF) scenario shows the consequences of a unilateral switch by the EU from the so-called Kyoto to Copenhagen obligations. The scenario assumes an emission reduction target of 15.5 % for the EU (i.e., 21 % reduction in the ETS and 10 % reduction in the non-ETS sectors according to EC (2010b, p.32) and a 4 % reduction target for the rest of Annex I (according to UNFCCC), all relative to 2004. The target for the EU is consistent with the 2009 Copenhagen Accord of 20 % reduction versus 1990 levels, but Annex I countries other than the EU are assumed to support only the reduction targets of the UNFCCC Kyoto Protocol. This scenario includes a central set of assumptions with respect to which alternative scenarios are defined. The main characteristics of the reference and alternative policy scenarios are summarized in Table 1. Each EU country imposes a domestic tax on the non-ETS sectors, whereas the remaining Annex I countries set a uniform carbon tax for all sectors. The revenue from the emission permits or carbon taxes are recycled back into the economies as a lump sum to households, keeping an equal yield constraint for governments.

**Table 1:** Policy scenarios

Characteristic/Scenario	BAU	REF	LOW	HIGH	BTA	CDM	CDM_NEW	OBA
Carbon reduction targets, in % relative to 2004								
EU	0	16	6	25	16	16	16	16
EU ETS	0	21	10	34	21	21	21	21
EU non-ETS	0	10	2	16	10	10	10	10
Rest of Annex I (A1)	0	4	4	4	4	4	4	4
Developing Countries (DC)	0	0	0	0	0	0	0	0
Allocation of emission allowances and carbon tax by sectors								
OBA	-	-	-	-	-	-	-	EU EITE
Auctioning & lump-sum recycling	-	EU ETS	EU ETS	EU ETS	EU ETS	EU ETS	EU ETS	EU ETS/non-EITE
Carbon tax & lump-sum recycling	-	EU non-ETS	EU non- ETS A1	EU non- ETS A1	EU non- ETS A1	EU non- ETS A1	EU non- ETS A1	EU non- ETS A1
Border carbon adjustments based on carbon content of traded goods								
Import tariffs	-	-	-	-	EU A1	-	-	-
Use of international carbon offsets								
Baseline emission level in DC	-	-	-	-	-	Before CO_2_ trading	After CO_2_ trading	-
Export premium for DC					-	yes	yes	-
Import premium for EU and A1					-	Yes	Yes	-
Limit as a % of reduction target in EU ETS					-	20 %	20 %	-
EU non- ETS					-	33 %	33 %	-
A1					-	100 %	100 %	-

*See text for explanation of acronyms

In the LOW scenario, pledges for the EU are lower, whereas the other assumptions remain unchanged. The scenario considers a hypothetical EU policy of 8 % CO_2_ reduction relative to 1990 levels, in line with the Kyoto Protocol commitments. We set emission limits at 90 % and 98 % of the 2004 levels for the ETS and non-ETS sectors, respectively, based on the PRIMES (i.e., a partial equilibrium model for the European Union energy markets that is widely used by the European Commission to analyze climate strategies) results (EC 2010b). This condition yields a total emission reduction of 6 % in the EU relative to 2004 levels.

In the HIGH scenario, the EU pledges are more ambitious than in the REF scenario. The HIGH scenario considers a possible future EU policy of 30 % CO_2_ reduction relative to 1990 levels to support high pledges of the UNFCCC Copenhagen Accord. EC (2010a) sets high pledges of 34 % and 16 % for the ETS and non-ETS sectors, respectively, versus 2005 levels. The border tax adjustment (BTA) scenario considers one of the contemplated instruments for reducing carbon leakage in the absence of a global climate agreement because the carbon intensiveness of exports is very high in many large developing countries. Border taxes are imposed by the EU and A1 on all imported products. The tax rate is based on the carbon content of the imported goods:

2 $$ {\mathrm{BT}}_{\mathrm{I},\mathrm{N},\mathrm{A}} = {\mathrm{P}\mathrm{C}}_{\mathrm{I},\mathrm{N}} - {\mathrm{P}}_{\mathrm{I},\mathrm{N}} = \mathrm{P}\_\mathrm{CO}{2}_{\mathrm{A}}\ *\ {\mathrm{c}}_{\mathrm{I},\mathrm{N}} $$

where BT_I_,_N_,_A_ is a tariff rate applied by region A on imported product I from region N, PC_I_,_N_ and P_I_,_N_ are the consumer price and producer price, respectively, of imported product i from region N, P_CO2_A_ is the domestic price of carbon in region A, and C _I_,_N_ is an emission intensity parameter for imported product I from region N. Future research could cover alternative definitions of tariff rates based on the carbon content in domestic production and a full BTA, which consists of both import and export adjustments. Additionally, alternative instruments, such as the taxation of international transport, could be evaluated. A combination of free emission permits in selected sectors and full auctioning in the remaining sectors is assumed in an output-based allocation scenario (OBA scenario), in which emission permits are grandfathered for EITE industries (chemical, minerals, metals, and paper). These sectors account for a relatively small share of the overall EU emissions and output (10 %), but unilateral emission limits raise concerns about their competitiveness. Thus, a free allocation of emission allowances to EITE industries may help the industries maintain their competitiveness. The allocation of free permits in the model is updated based on sectoral outputs, and it covers 100 % of the emissions in the eligible sector. The allocation is handled as an implicit production subsidy, contingent upon firms’ production decisions:

3 $$ {\mathrm{OS}}_{\mathrm{I}} = \mathrm{P}\_\mathrm{CO}2\ *\ {\mathrm{b}}_{\mathrm{I}}\ /\ {\mathrm{Q}}_{\mathrm{I}} $$

where OS_I_ is the subsidy rate for sector I in the EU, Q_I_ is the domestic output of product I, P_CO2 is the carbon price for ETS sectors, and b_I_ is an emission parameter based on sectoral carbon emissions in 2004 increased by emission pledges for 2020. Thus, the revenues of sectors obtaining free allowances are increased by (P_CO2 * b_I_). Additional allowances are granted if production increases, and the carbon price constitutes an incentive for reducing emission intensity. The welfare loss via production subsidies will be very small if the EITE sectors do not have a considerable share of the overall emissions and output.

Finally, we apply two scenarios of international carbon offsets: the CDM and CDM_NEW scenarios. While the former assumes that the offset subtracts from a hypothetical emission, the latter assumes that the hypothetical emission is its result. Thus our simulation experiment includes two cases, where the baseline emission level for non-Annex I countries is determined before and after the mechanism is implemented. A CDM scenario coincides with the REF scenario, but inter-regional emission trading is allowed; the ETS and non-ETS sectors can purchase up to 20 % and 33 % of their emission reduction requirements, respectively (EC 2008, 2009). No limits for international trading with DC are applied to other Annex I countries. Equalization of reduction costs in the EU and A1 regions, with a cost of acquiring offset units, indicates that the difference between the reduction cost in the DC region and the EU or A1 region is included in export and import premiums, as illustrated by the following equation:

4 $$ \mathrm{P}\_\mathrm{CO}{2}_{\mathrm{A}} = \mathrm{P}\_\mathrm{CO}{2}_{\mathrm{N}} + {\mathrm{PX}}_{\mathrm{N}} + {\mathrm{PM}}_{\mathrm{A}} $$

where P_CO2_A_ is the carbon price in region A (EU or A1) with emission targets, P_CO2_N_ is the marginal abatement cost in region N without emission obligations, PX_N_ is the export premium for the government in region N, and PM_A_ is the an import premium for the government in region A. Therefore, the total premium is divided between exporting and importing regions. If there is no export limit for the emission reduction in region N (i.e., supply in DC is greater than demand in the EU and A1), then PX_N_ = 0 and the whole premium is taken over by the government in region A. If the export limit for the emission reduction is not greater than the demand for emission reduction by region A, then PX_N_ > 0 and PM_A_ decreases. We consider the second case, in which the export limit for emission reduction in DC is not greater than the demand for emission reduction by the EU.^6^

The CDM_NEW scenario differs from the CDM scenario in which the initial emission level (before inter-regional emission trading) in DC is the BAU level. In this situation, the DC region indeed participates in the global climate action. However, because the DC region has no binding carbon reduction target relative to the BAU level, the region has an incentive to inflate its emission level before trading with the EU or A1 regions. Thus, in the CDM_NEW scenario, we assume that after the completion of the carbon offset transaction, emissions in DC reach the BAU level.

## Simulation results

**Fig 1:**
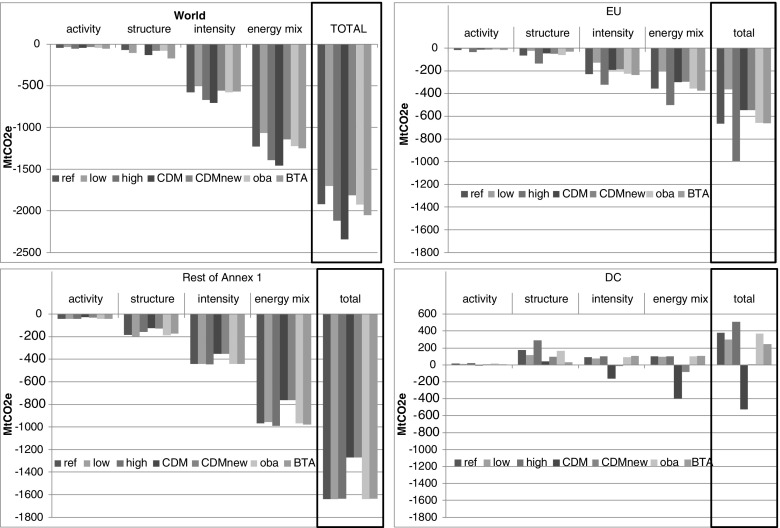
Decomposition of the change in carbon emissions into four effects (activity, structure, intensity, and energy mix) by region, relative to BAU*

The largest reduction in carbon emissions is achieved if all regions participate in the climate action (CDM scenario), whereas the reductions delivered by the remaining scenarios are lower (Fig. 1). Relative to the BAU levels, the reduction of global emissions is between 5 % (LOW) and 7 % (CDM). The DC countries increase their emissions in all scenarios, except in CDM and CDM_NEW. Changes in carbon emission may be studied by quantifying the impact of several factors. We use additive decomposition with the LMDI (Logarithmic Mean Divisia Index) method (Ang 2005), where four factors are considered:

1. Activity effect, which reflects overall regional activity (∑_I_Q_I,R_)
2. Structure effect, which reflects the activity mix (Q_I,R_/∑_I_Q_I,R_)
3. Intensity effect, which reflects sectoral energy intensity (C_F,I,R_/Q_I,R_)
4. Energy mix effect, which reflects the fuel mix (C_F,I,R_/∑_F_C_F,I,R_)

where Q_I_,_R_ is an output in sector I in region R and C_F_,_I_,_R_ is the consumption of fuel F by sector I in region R.

The activity effect is insignificant in all policy scenarios. This finding is consistent with the macroeconomic results (Table 2) and shows that the welfare/GDP deviation from the BAU is less than 1 %. Only in the HIGH scenario is the decline in the EU’s GDP at 1.7 %, but this value can still be regarded as fairly small. Second, the largest emission reduction in the EU and A1 results is induced by a switch toward less carbon-intensive fuels. This switch is possible through a dramatic phase-out of coal combined with higher imports of electricity, leading to increased emissions in DC, which is the essence of carbon leakage. Third, the intensity effect is responsible for approximately one-quarter of emission reductions in the EU and A1 countries due to a shift toward less energy-intensive production technologies. Finally, changes in economic structures contribute approximately 10 % to a decline in emissions in the EU and A1 countries. These economies have already completed a major shift toward services that are less carbon-intensive than those used in industry, and this effect could not be large in the future. Due to the structure effect in the BTA scenario, DC’s emissions are being constrained compared to those in other scenarios. Although the magnitude is relatively small, this result suggests that import taxes based on carbon content may provoke a structural reallocation of resources in DC economies toward less carbon-intensive sectors.

**Table 2:** Macroeconomic results by scenario [% deviation from BAU*]

Scenarios	Welfare			GDP			Unemployment rate [pp deviation from BAU]			
	EU	A1	DC	EU	A1	DC	EU	A1	DC	
LOW	−0.1	−0.3	−0.1	−0.2	−0.5	−0.05	0.1	0.2	0.01	
REF	−0.5	−0.4	−0.2	−0.7	−0.5	−0.1	0.3	0.2	0.02	
HIGH	−1.2	−0.4	−0.3	−1.7	−0.5	−0.2	0.6	0.2	0.04	
CDM	−0.3	−0.3	−0.1	−0.5	−0.4	−0.1	0.2	0.1	0.05	
CDMnew	−0.3	−0.3	−0.04	−0.5	−0.4	−0.03	0.2	0.1	0.03	
OBA	−0.4	−0.4	−0.2	−0.7	−0.5	−0.1	0.3	0.2	0.02	
BTA	−0.3	−0.4	−0.5	−0.7	−0.5	−0.2	0.3	0.2	0.02	
Scenarios	Trade balance			Carbon price [USD 2004 per t CO_2_]				Electricity price		
-				EU	EU non	-				
-	EU	A1	DC	ETS	ETS	A1	DC	EU	A1	DC
LOW	−0.1	−1	2	21	21	30	-	5	13	−0.6
REF	−7	−0.4	2	49	96	31	-	11	13	−0.8
HIGH	−18	0	3	118	197	32	-	24	13	−0.9
CDM	0	1	−2	36	58	20	2	8	9	1.3
CDMnew	−0.2	1	−2	36	58	20	1	8	9	0.0
OBA	−7	−0.4	2	50	89	31	-	11	13	−0.8
BTA	−37	−2	2	53	104	31	-	12	13	−1.0

* See Table 1 for scenario descriptions

Table 2 summarizes the most important outcomes. Welfare negatively reacts to the emission ambition level because no benefits from emission reduction were considered. These adverse effects affect the developing countries and are a result of repercussions observed in the countries’ importing partners. In Annex I regions, both welfare and output losses and unemployment rate increases are manageable. Only in the HIGH scenario is the loss in the EU’s GDP greater than 1 %. However, the EU loses its competitiveness, and the trade balance goes down considerably. At the same time, the A1 countries improve their trade balance with the higher emission target in the EU.

Higher carbon reductions are reflected in higher carbon prices, which drive the relative price increases of electricity in the EU and A1. The shares of net fuel cost and carbon cost in the total cost of electricity production are 19 % and 6 % in the EU and 31 % and 13 % in A1, respectively, in scenario BAU. In the REF scenario, the slightly faster growth of the price of electricity is driven by the carbon intensity of power generation, which is higher in A1 than in the EU. In the HIGH scenario, the electricity price grows faster in the EU than in A1 because the share of carbon cost becomes greater than the fuel purchase cost. Lower demand for energy in A1 and the EU generates a lower price for fuels, and electricity production in DC becomes cheaper.

The carbon price in A1 slightly increases, regardless of the constant emission target in all three scenarios, as a result of a weaker economy in the EU. The carbon price is higher in A1 than in the EU in the LOW scenario because there are higher emission targets for A1 than for the EU relative to the BAU level. The reduction target relative to 2004 levels (4 % and 6 % in A1 and the EU, respectively; see Table 1) corresponds to emission reductions of 15 % and 9 % relative to the BAU level as a result of higher energy consumption in A1 according to the forecast by IEA (International Energy Agency). The shadow carbon price in the non-ETS sectors is similar to that in the ETS sectors in the LOW scenario but significantly differs in other scenarios. This result suggests that the marginal abatement cost is similar for ETS and non-ETS sectors in the LOW scenario. However, the target distribution proposed by the European Commission for the second commitment period (scenario REF) is far from efficient because the marginal abatement cost is significantly higher in non-ETS sectors. Thus, the target for non-ETS sectors should be decreased relative to that for ETS sectors.

**Fig 2:**
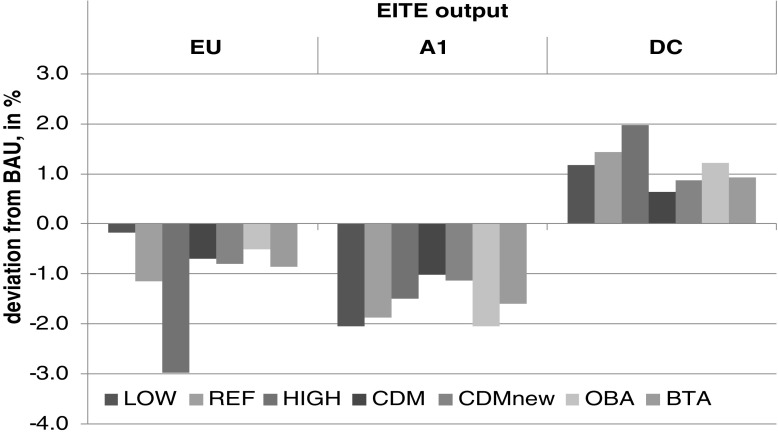
Output in sectors exposed to a risk of carbon leakage (deviation from BAU* in %)

Our next exercise addresses potential competitiveness losses implied by unilateral climate actions. OBA and BTA protect EU markets from imports, which helps to slow down the decrease in welfare and output. Additionally, CDM allows for improvement because targeted emissions are reached in a less costly manner. From the EU perspective, the OBA scenario is attractive because EITE sectors’ output is the highest (Fig. 2). From the A1 perspective, OBA is the least favorable because an output from the EU EITE sectors is not reallocated to A1 and DC. The welfare effect for DC is most detrimental under the BTA regime.

Unilateral actions of the EU may change the global emissions only marginally. Under the most ambitious scenario (HIGH), the global emissions are 7 % below the BAU level. Scenarios REF, OBA, and BTA yield the same effect on global emissions, but the leakage rates differ (Table 3). According to the definition provided in Section 2, the carbon leakage rate required for the policy to move from LOW to REF is 22 %, which is moderate. The rate can be slightly reduced to 19 % if domestic producers in the EITE sectors are protected through free emission allowances (OBA scenario). Carbon taxes on imported goods (BTA scenario) appear to be a much more effective anti-leakage measure because the CL rate becomes negative under such taxes. The effect is similar in the CDM scenario; the CL rate is negative because the reduction in DC and A1 doubles the reduction in the EU. It is likely that DC will behave according to scenario CDM_NEW rather than the CDM scenario. The CDM_NEW results in an increase in the CL rate to 40 % due to expanding emissions in the A1 countries, whereas emissions remain at the BAU level in the DC region. The difference between the negative CL rate in the CDM scenario and the positive CL rate in the CDM_NEW scenario is striking.

**Table 3:** Global CO_2_ emissions and carbon leakage rate

Scenarios*	BAU	LOW	REF	HIGH	CDM	CDMnew	OBA	BTA
Global emissions								
% of BAU	100	95	94	93	93	95	94	94
% of LOW	105	100	99	98	98	100	99	99
Leakage rate								
Relative to LOW								
Region A** = EU								
(our concept)	-	-	22	28	−200	40	19	−16
Relative to BAU								
Region A = EU								
(wrong concept)	-	−368	−177	−107	−306	−218	−181	−195
Relative to LOW								
(wrong concept)	-	-	22	28	503	181	19	−16
Relative to BAU								
Region A = EU + A1								
(common, but wrong concept)	-	14	16	18	−28	0	15	10

* See Table 1 for scenario descriptions

**Region A means a region with a GHG abatement program

The CL rate increases from 22 % to 28 % with the increased EU target. This result is comparable with that obtained for the REF scenario only because other scenarios do not comply with the ‘everything else being constant’ assumption. When we change several attributes between scenarios, it does not make sense to compare CL rates. There are two other details that make our concept of CL different from that adopted in the mainstream literature. We relate CL to the LOW scenario, whereas the usual starting point is the BAU scenario. The majority of Annex I countries have adopted the UN FCCC Kyoto Protocol, which assumes some low-carbon abatement effort; therefore, the LOW scenario is a more appropriate benchmark than BAU for CL analysis.^7^

The second detail is related to the distinction between abating (A) and non-abating (N) regions. With the LOW scenario as a benchmark, the countries with less ambitious abatement targets (A1) and the countries with no binding abatement targets (DC) are grouped together because both regions may be a destination for emissions leaking from the region with the more ambitious climate policy (i.e., EU). If we ignore this distinction, then A1 is interpreted as one of the regions that undertake some climate action (regardless of whether it was historically accomplished or planned for the future) and becomes an abating region. In this case, the problem of interpretation arises in scenarios with CDM, in which carbon leakage rates are enormous. Such a result is difficult to accept because the idea of CDM is to reduce CL, not to increase it. Additionally, the CDM scenario cannot generate a higher CL rate than that generated in the CDM_NEW scenario according to the definitions of these scenarios.

Thus, our results for CL are not directly comparable with those reported in the mainstream literature. If we apply a common definition (i.e., the baseline scenario is BAU and the non-abating region is only DC), then the results will be underestimated in most cases. When CL is negative, these results are overestimated (scenarios CDM and BTA). Thus, traditional concepts of CL do not provide a true picture of reality. For example, the BTA scenarios allow for only a slight reduction in CL according to the common definition. However, the scenarios will eliminate CL according to our definition.^8^

## Sensitivity analysis

The sensitivity analysis shows that the results generated by the CGE models may be assumption-driven and should be interpreted with caution. As noted by Hillberry and Hummels (2012), it is common when calibrating CGE models to adopt trade elasticities from the literature. For a sensitivity analysis, we divide or multiply the benchmark parameter values from the literature by 2. The results, with one exception, show that unilateral European climate action is not detrimental to global emissions because CL < 100 %. The exception is related to the elasticity of substitution between imported regions, whose doubling increases the value of CL to 102 %. Therefore, we cannot ensure that European policy is not detrimental to the environment. The details of our sensitivity analysis are presented in a submitted paper (Kasek et al. 2012).

## Conclusions

After a careful review of existing concepts of carbon leakage, we provided an alternative definition emphasizing what must be kept constant and consistently applied it in a CGE framework to assess the EU unilateral carbon abatement commitments in 2020. Unilateral carbon abatement policies can be counter-productive because a large part of emissions reduced in Annex I countries may be offset by an increase in emissions in the rest of the world. Our policy simulations suggest that more stringent abatement commitments by the EU not only lead to a higher CL rate but also translate into higher welfare or output losses for all regions. The EU welfare effects can be mitigated by anti-leakage measures, but the situation becomes a zero-sum game if the corresponding effects in the DC region are considered. International carbon offsets could be part of a solution if the DC countries determine their baseline emissions before CDM transactions.

Only a global action could result in global climate protection, and from this perspective, any regional policies prove to be insufficient. According to EIA (2009), the EU will be responsible for only 11 % of global emissions in 2020; hence, its unilateral actions are doomed to fail in solving the global problem. However, even if the United States decided to participate in the global climate protection effort, as long as emerging economies such as China, India, Russia, or Brazil do not reduce emissions in absolute terms, there is little chance of meeting global targets for stabilizing the CO_2_ concentration in the atmosphere. Using the LMDI approach, we decomposed the change in emissions by region into their four major drivers. The largest emission reduction is due to a switch toward less carbon-intensive fuels, whereas the activity effect is insignificant.^9^ Thus, investments can be encouraged by emission quotas.

Simulation results crucially depend on the technical assumptions made. Some parameters affect not only the magnitude but the sign of the carbon leakage rate as well. CGE models are powerful tools for policy analyses, but their results require a careful validation of the underlying technical assumptions. The PRIMES is an official model used by the EC, but because the model’s details are hidden, its results cannot be replicated by other scholars. However, technical assumptions adopted in such models are of critical importance for policy simulations. We identified a list of parameters that affect not only the magnitude but also the sign of the carbon leakage rate. Changing parameter values suggests that, as a result of a unilateral action by the EU, other countries may either increase or decrease their carbon emissions. A careful validation of these assumptions is necessary before the policy simulations may support the evidence-based policy recommendations. Widely used by the EU institutions, the PRIMES model simulates the carbon price for ETS sectors to be 25 and 39 EUR for 8 % and 20 % reductions, respectively. We obtained values of 19 and 43 EUR, respectively. The historic average of the ETS allowance price for the first commitment period was EUR 15, but it is currently EUR 6. Hence, compared to the PRIMES estimate, our estimated value is closer to the real value.

A recent overview of climate policies (Tol 2013) does not emphasize a need for a global agreement because, indeed, this issue is rarely addressed in the academic literature. Our analysis stresses that non-global agreements compromise both environmental effectiveness and economic efficiency. Any unilateral initiatives are insufficient and insignificant to the magnitude of the problem, and they might lead to cumulative climate damages (see, e.g., Stavins 2013, Ritter and Schopf 2014). The value added by the paper can be summarized as follows: First, we show that the current concept of carbon leakage used in the literature provides an incorrect view of the leakage problem. Second, we demonstrate sources of emission reduction via decomposition analysis. Third, we explain why a clean development mechanism may fail. Fourth, we show that the current distribution of emission permits among sectors adopted by the European Commission is inefficient. Finally, we explain why mitigation strategies for climate change should be global rather than local.

## Footnotes

1 For example, Gerlagh and Kuik (2007) relate modeling results from the literature to some elasticities to explain the relationship between parameter choice and carbon leakage. However, the authors do not address the fact that there may be other driving factors as well. The carbon leakage results depend on which regions are defined as those that undertake an abatement program and what is the baseline scenario that serves as a benchmark. If the baseline case assumes no reduction target, then the carbon leakage rate for a 20 % reduction target, for example, will be smaller than the carbon leakage rate for the same reduction target calculated in relation to a baseline with a non-zero reduction target.

2 Negative values (i.e., emission reduction in N corresponding to an increased carbon abatement target adopted in A) may arise as a result of complex processes that take place in the world economy following an increased abatement effort in one region. For example, such a situation occurs if the transition of A to a low-emission economy induces strong technological progress that is then adopted in N. In this case, increased global output could be accompanied with less pollution globally. Positive values (i.e., emission increase in N corresponding to an increased abatement target adopted in A) may result from moving production to where it is not constrained by environmental standards.

3 This can be expected when production moves to locations with higher carbon intensity (i.e., producers in N are less carbon efficient than producers in A) or where positive long-run effects related to technological progress are weaker. Under certain circumstances, the CL rate can be zero or negative, which means that there is no increase in emissions in N. For instance, this situation may arise when border tax adjustments are implemented.

4 The model is described in greater detail in Kasek et al. (2012).

5 No emission limits are applied. In the baseline scenario, world energy-related carbon dioxide emissions grow from 26 Gt in 2004 to 36 Gt in 2020. World consumption of oil grows by 25 %, whereas coal and gas consumption grow by 40 % and 60 %, respectively. Future world oil prices increase significantly by 185 % relative to 2004 levels. The world prices of gas and coal rise by 40 % and 10 %, respectively. GDP in the EU and other Annex I countries will grow by 40 %, but they will grow in DC by 130 %. Total energy-related CO_2_ emissions grow by 40 % (by 4 % in the EU, by 13 % in the rest of Annex I, and by 70 % in DC).

6 This implies that the export premium for DC and the import premium for the EU are positive. No limit on international carbon offsets for A1 drives the import premium for A1 to zero.

7 In particular, the current policy choice for the EU is not between doing nothing (BAU) and adopting the 20-20-20 package (REF). Indeed, the choice is between the LOW and REF or between the REF and HIGH scenarios. If we ignore the choice, then the CL rate becomes negative for all scenarios; therefore, we may send the wrong signal to policy makers.

8 The BTA increases production in the abating region (then CL goes down), which increases the demand for carbon emissions while the emission target remains the same as in the REF scenario (then the carbon price increases, as shown in Table 2).

9 Lockwood and Thomas (2002) provide theoretical proof for such behavior. Although it may be desirable to move immediately to reduce capacity in an industry to some level, this situation does not represent an equilibrium because either firm would prefer to have the other one reduce capacity while retaining its own capacity.
